# Clusterin overexpression in mice exacerbates diabetic phenotypes but suppresses tumor progression in a mouse melanoma model

**DOI:** 10.18632/aging.202788

**Published:** 2021-03-10

**Authors:** Christina Cheimonidi, Ioannis N. Grivas, Fabiola Sesti, Nadia Kavrochorianou, Despoina D. Gianniou, Era Taoufik, Fotis Badounas, Issidora Papassideri, Federica Rizzi, Ourania E. Tsitsilonis, Sylva Haralambous, Ioannis P. Trougakos

**Affiliations:** 1 Department of Cell Biology and Biophysics, Faculty of Biology, National and Kapodistrian University of Athens, Athens 15784, Greece; 2 Inflammation Research Laboratory, Department of Immunology, Transgenic Technology Laboratory, Hellenic Pasteur Institute, Athens 11521, Greece; 3 Laboratory of Cellular and Molecular Neurobiology-Stem Cells, Hellenic Pasteur Institute, Athens 11521, Greece; 4 Dipartimento di Medicina e Chirurgia, Universita di Parma, Parma 43125, Italy; 5 Istituto Nazionale Biostrutture e Biosistemi (I.N.B.B.), Roma 00136, Italy; 6 Department of Animal and Human Physiology, Faculty of Biology, National and Kapodistrian University of Athens, Athens 15784, Greece

**Keywords:** cancer, chaperone, clusterin, diabetes, proteostasis

## Abstract

Clusterin (CLU) is an ATP-independent small heat shock protein-like chaperone, which functions both intra- and extra-cellularly. Consequently, it has been functionally involved in several physiological (including aging), as well as in pathological conditions and most age-related diseases, e.g., cancer, neurodegeneration, and metabolic syndrome. To address CLU function at an *in vivo* model we established CLU transgenic (Tg) mice bearing ubiquitous or pancreas-targeted CLU overexpression (OE). Our downstream analyses in established Tg lines showed that ubiquitous or pancreas-targeted CLU OE in mice affected antioxidant, proteostatic and metabolic pathways. Targeted OE of CLU in the pancreas, which also resulted in CLU upregulation in the liver likely via systemic effects, increased basal glucose levels in the circulation and exacerbated diabetic phenotypes. Furthermore, by establishing a syngeneic melanoma mouse tumor model we found that ubiquitous CLU OE suppressed melanoma cells growth, indicating a likely tumor suppressor function in early phases of tumorigenesis. Our observations provide *in vivo* evidence corroborating the notion that CLU is a potential modulator of metabolic and/or proteostatic pathways playing an important role in diabetes and tumorigenesis.

## INTRODUCTION

Clusterin (CLU) is a secreted glycoprotein being expressed in various tissues including liver, brain, ovary, testis, heart, and blood vessels, and has been functionally involved in many different physiological and pathological processes [1, 2]. CLU acts like a stress-activated, extracellular ATP-independent small heat shock-like chaperone, whose levels are elevated during aging, cancer, and neurodegenerative disorders [3]; recent studies have also highlighted a role for CLU in intracellular proteostasis [4] to suppress proteotoxicity.

Reportedly, CLU is implicated in pancreatic physiology, as well as in metabolic regulation and metabolic disorders. Specifically, it was shown that partial pancreatectomy in CLU knockout (KO) mice did not result in complete regeneration of the organ and β-cell production was incomplete [5], while transfection of pancreatic cells with CLU cDNA increased cell proliferation and differentiation [6]. Furthermore, CLU is highly expressed in pancreatic tubular complexes in hypertensive rats during spontaneous pancreatitis and enhanced islet regeneration [7]. Serum CLU levels are elevated in patients with type 2 diabetes (T2D) and correlate positively with blood glucose (GLU) levels [8]; moreover, CLU gene polymorphisms have been associated with T2D and a strong correlation between serum CLU levels and insulin (INS) resistance markers was discovered [9]. Nonetheless, although these studies indicate a link between CLU and diabetes the underlying mechanisms remain largely unknown.

CLU is also implicated in all stages of cancer, i.e., progression, promotion, metastasis and chemoresistance acquisition [1]. There is also evidence that CLU inhibition at late stages of tumor evolution can have beneficial effects in therapy, whereas at early stages increased levels of CLU likely suppress tumorigenesis [10]. Intracellularly, CLU was (among others) found to stabilize the cytosolic Ku70-Bax complex, inhibiting thus pro-apoptotic Bax to activate apoptosis [11].

Herein, we report the establishment of CLU overexpressing transgenic (Tg) mice; either ubiquitously or with pancreas-targeted expression. We show that targeted CLU overexpression (OE) in the pancreas increased basal GLU levels in the circulation and exacerbated diabetic phenotypes. Also, by employing a syngeneic melanoma mouse model in CLU Tg (ubiquitous OE) mice we found that ubiquitous CLU OE delayed melanoma tumor cells growth, indicating a likely tumor suppressor function in early phases of tumorigenesis.

## RESULTS

### Ubiquitous or pancreas-targeted CLU OE in mice affects proteostatic and metabolic pathways

**Figure 1:**
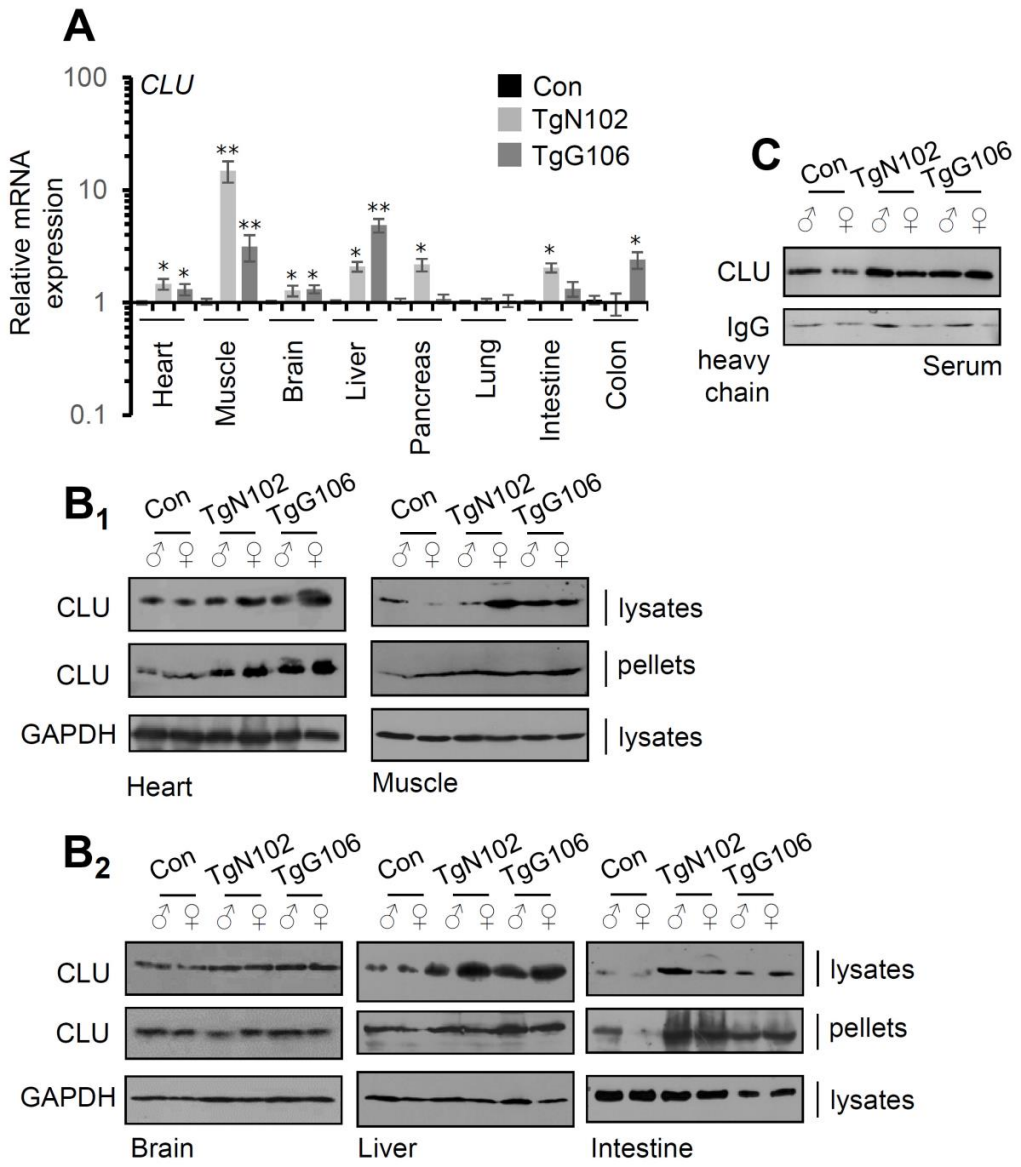
**CLU is overexpressed in tissues of the TgN102 and TgG106 (ubiquitous CLU OE) mice.** (**A**) Relative *clu* mRNA expression levels in the heart, muscle, brain, liver, pancreas, lung, intestine, and colon of TgN102, TgG106 lines and control (littermate non-Tg) animals. (**B**) Representative immunoblot analyses in shown tissue samples [whole cell lysates and cell membranes (pellets)] from Tg or control animals probed with a CLU antibody; GAPDH probing was used as a reference. (**C**) Immunoblot analyses of CLU expression levels in serum of shown Tg or control animals; IgG probing was used as loading reference. Error bars, ± SD (n=4 per mouse genotype); *P<0.05; **P<0.01.

**Figure 2:**
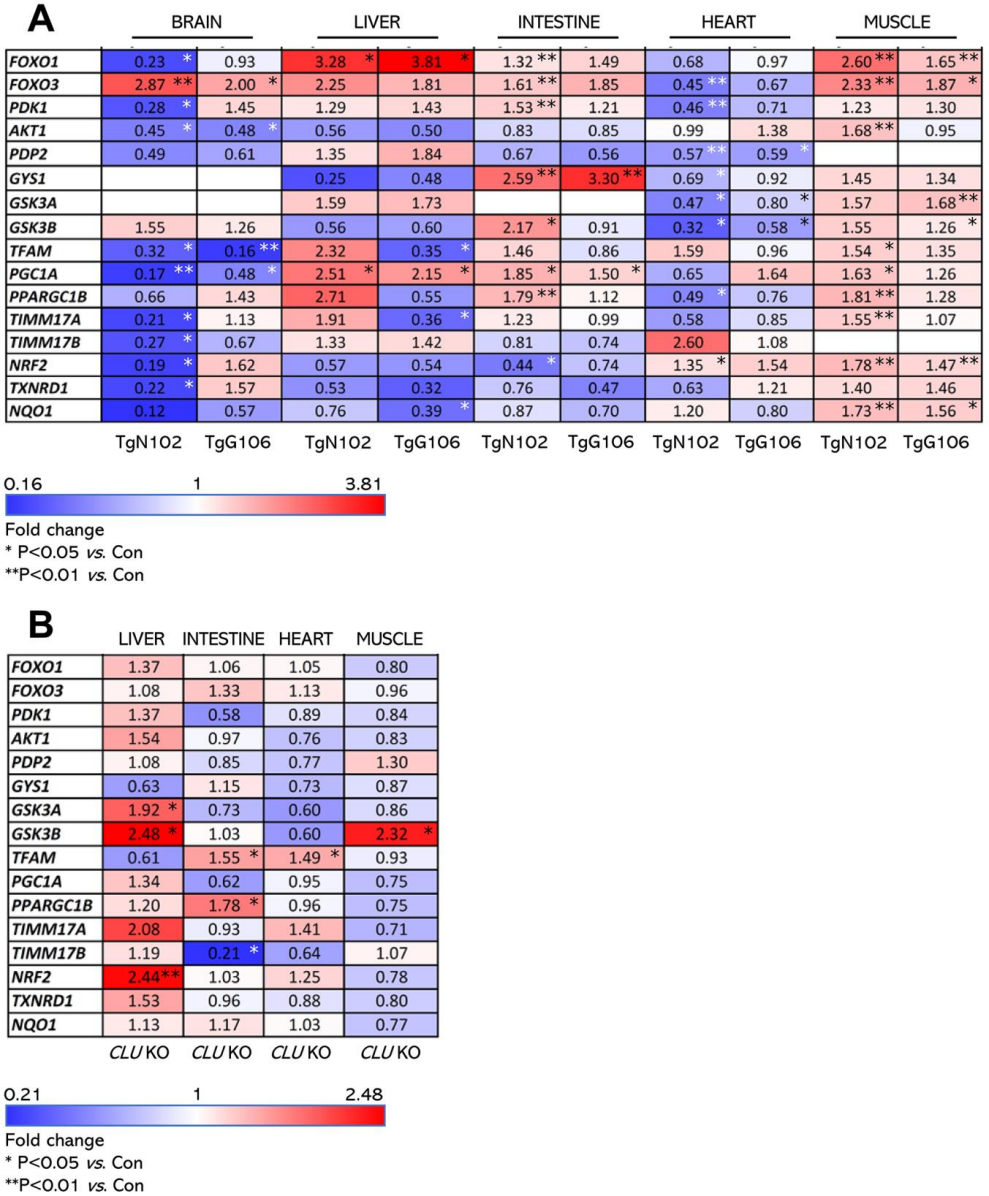
**Expression levels (*vs*. controls) of antioxidant, mitochondrial and metabolic genes (heat map) in isolated shown tissues of TgN102 and TgG106 (ubiquitous CLU OE) mice or in CLU KO mice.** (**A**) Heat map indicating relative expression levels of shown genes in isolated brain, liver, intestine, heart, and muscle tissues of TgN102 and TgG106 (ubiquitous CLU OE) *vs*. control (Con; littermate non-Tg) mice. (**B**) Heat map of shown genes expression in isolated liver, intestine, heart, and muscle tissues of *CLU* KO *vs*. control mice. *P<0.05; **P<0.01 (Tg or KO mice *vs*. Con); additional statistical analyses (i.e., Pearson Correlation *r* and *F* significance) are shown in Supplementary Tables 1, 2.

Our gene and protein expression analyses showed that both the TgN102 and TgG106 lines carrying the human (h)β*actin*-*clu* Tg express higher *clu* levels (*vs*. non-Tg littermates) in the heart, muscle, brain, liver, and intestine (significant at the TgN102 line) tissues (Figure 1A). These findings were further verified at the protein expression level (in the brain CLU expression was moderately increased) in both the soluble cell fraction (cell lysates) and in cell membranes (cell pellets) of isolated tissues (Figure 1B). Consistently, CLU levels increased in the sera of Tg animals (Figure 1C). Gene expression analyses in a panel of antioxidant, metabolic and mitochondrial genes in CLU overexpressing tissues from the TgN102 and TgG106 mouse lines revealed similar expression patterns in the two lines for most tissues analyzed (Figure 2A; Supplementary Table 1); these similarities were more intense in liver, intestine, and the muscle. Also, gene expression patterns amongst tissues were similar (for both Tg lines) mostly between liver and muscle (Figure 2A; Supplementary Table 1). Parallel gene expression analyses in *CLU* deficient mice (C57BL/6-*clu* KO; herein indicated as CLU KO) revealed a rather tissue-specific pattern with most similarities observed between intestine and heart and to a lesser extend (as in Tg lines) between liver and muscle (Figure 2B; Supplementary Table 2). In a tissue specific pattern, the majority of analyzed genes tended to be upregulated (*vs*. Con; non-Tg littermate mice) in the muscle of Tg CLU overexpressing mice (Figure 2A); interestingly enough, these were suppressed in *CLU* KO mice indicating that alterations in CLU expression levels likely impacts on genomic responses (Figure 2A, 2B). In support, the expression of the antioxidant responses-related transcription factor *nrf2* and of its target *txnrd1* and *nqo1* genes seem to be differentially regulated in CLU OE and *CLU* KO mice as it tends to be downregulated in the liver and intestine of *CLU* OE mice and to be induced in the same tissues in *CLU* KO mice (Figure 2A, 2B); notably the opposite regulatory readout for these genes was noticed in the muscle (Figure 2A, 2B). Regarding metabolic/mitochondrial genes, *tfam* and *pgc-1a* were suppressed in the brain but tended to be induced in other tissues, while *foxo3* was downregulated only in the heart (Figure 2A). Given that *pgc-1a* was downregulated in the intestine and muscle of *CLU* KO mice (Figure 2B) we hypothesize that its expression levels are likely directly modulated in these tissues by CLU expression levels. These findings suggest tissue-dependent effects after CLU OE that can possibly be attributed to (among others) differential tissue-dependent metabolic demands.

**Figure 3:**
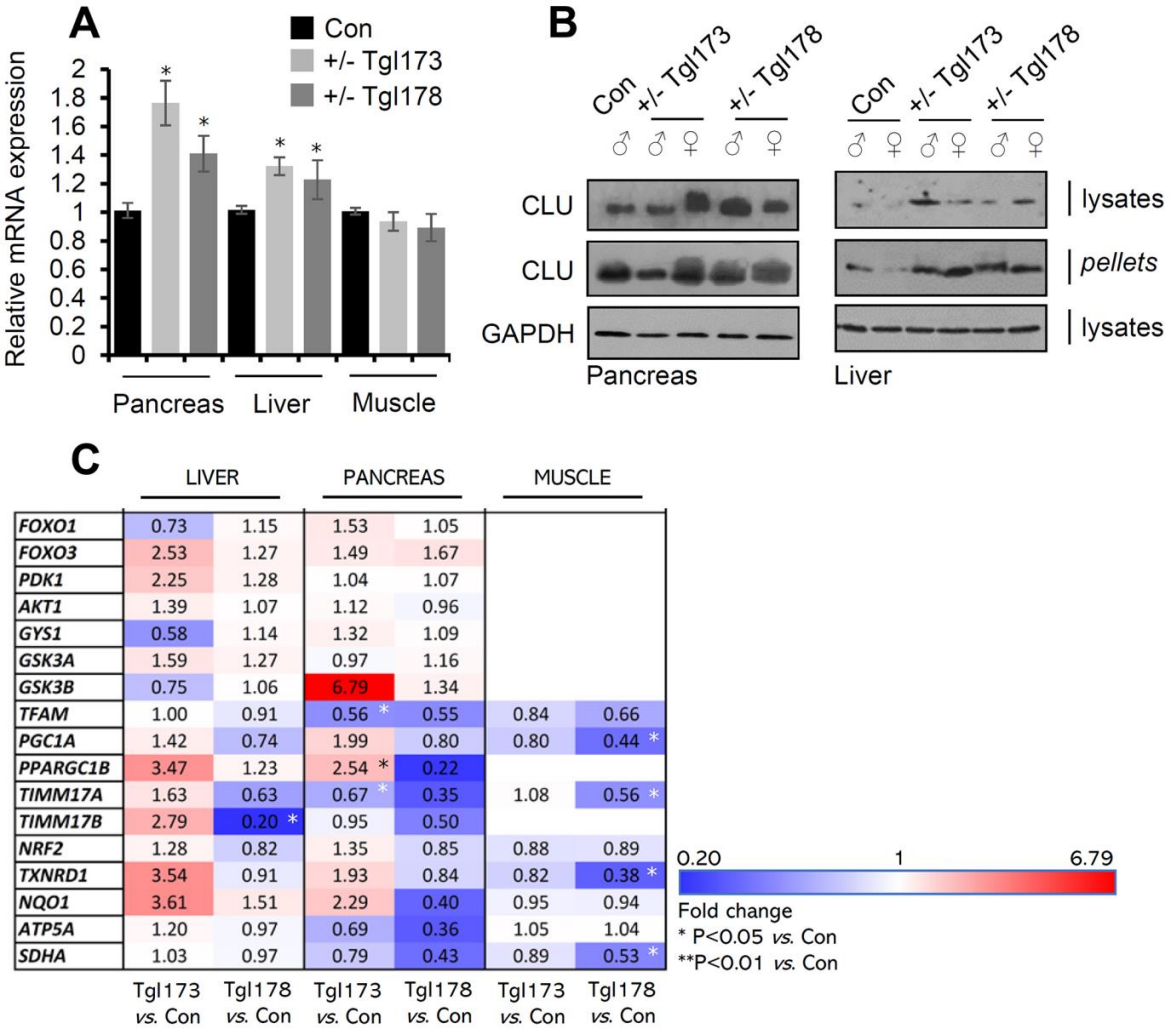
**CLU is overexpressed in the pancreas and liver of TgI173, TgI178 (pancreas-targeted CLU OE) mice.** (**A**) Relative *clu* mRNA expression levels (*vs*. control; littermate non-Tg animals) in the pancreas, liver, and muscle of TgI173 (+/-), TgI178 (+/-) animals. (**B**) Representative immunoblot analyses of shown Tg (or not) animals’ tissues samples [whole cell lysates and cell membranes (pellets)] probed with a CLU antibody; GAPDH was used as a reference. (**C**) Heat map indicating relative expression levels of shown genes in isolated liver, pancreas, and muscle tissues of TgI173 and TgI178 Tg *vs*. control (Con; littermate non-Tg) mice. Error bars ± SD (n=4-5 per mouse genotype); *P<0.05 (Tg lines *vs*. control). Statistical analyses (i.e., Pearson Correlation *r* and *F* significance) of data shown in Figure 3C are reported in Supplementary Table 3.

Our attempts to establish pancreas-specific CLU overexpressing animals carrying the *pdx-1-clu* transgene resulted in two Tg heterozygous lines (TgI173, TgI178) carrying the Tg; notably, we could not establish homozygous animals because of increased embryonic lethality. We found that *clu* mRNA and CLU protein expression levels were elevated in the pancreas of the TgI173 and TgI178 lines, and also in liver (Figure 3A, 3B) indicating CLU OE-mediated systemic effects. CLU OE in isolated pancreas and liver tissues was evident both in the soluble cell fraction (cell lysates) and in cell membranes (cell pellets) (Figure 3B). Gene expression analyses in metabolic tissues, i.e., pancreas, liver and muscle of TgI173 and TgI178 *vs*. non-Tg littermate mice, revealed some level of heterogenicity amongst the two Tg lines (Figure 3C; Supplementary Table 3); yet a notable trend for several metabolic genes downregulation was found in the muscle and pancreas (most evident in the Tgl178 line) tissues. On the other hand, for most genes assayed in the liver we noted a tendency for higher expression levels, with *foxo3* showing a similar pattern of upregulation in both the liver and the pancreas of the Tgl173 and Tgl178 lines (Figure 3C). Thus, ubiquitous, or pancreas-targeted CLU upregulation in mice alters basal expression levels of antioxidant, proteostatic, mitostatic and metabolic genes.

### Pancreas-targeted CLU OE exacerbates diabetic phenotypes

**Figure 4:**
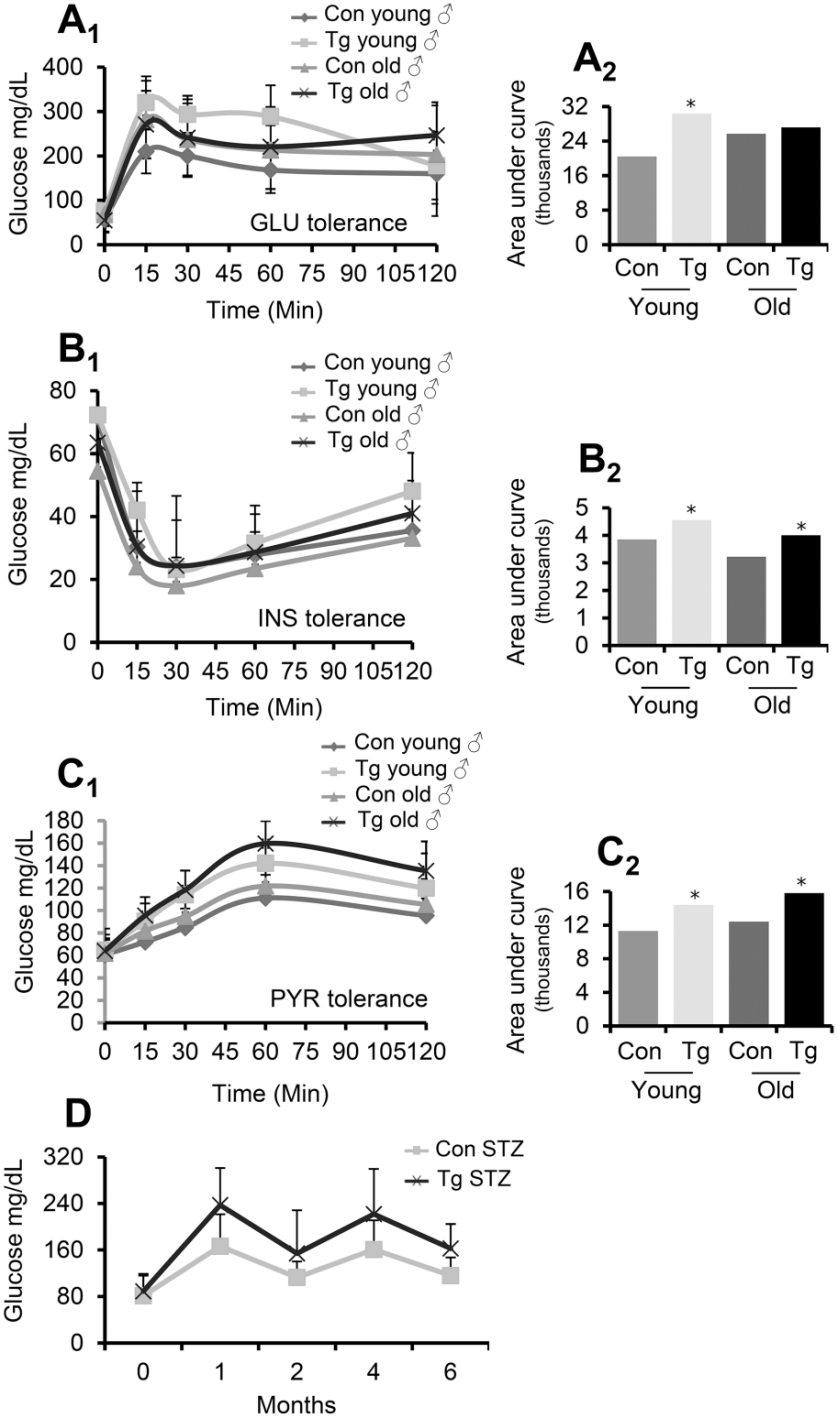
**Pancreas-targeted CLU OE induces GLU, INS and PYR decreased tolerance.** (**A**) GLU tolerance curve (**A_1_**) and area under the curve (**A_2_**, calculated from the sum of the areas of the different trapeziums formed) in shown animal groups; GLU levels were measured before and after (15, 30, 60 and 120 min) GLU injection. (**B**) INS tolerance test. Shown is GLU tolerance curve (**B_1_**) and area under the curve [**B_2_**, calculated as in (**A_2_**)] in indicated animal groups; GLU levels were measured before and after (15, 30, 60 and 120 min) INS injection. (**C**) PYR tolerance test. Shown is GLU tolerance curve (**C_1_**) and area under the curve [**C_2_**, calculated as in (**A_2_**)] in indicated animal groups; GLU levels were measured before and after (15, 30, 60 and 120 min) PYR injection. (**D**) GLU levels following STZ administration in control (Con STZ) and Tg (Tg STZ) mice. GLU levels were measured during the whole duration of the experiment (6 months). Con; young or old littermate non-Tg male animals; Tg; young or old pancreas-targeted CLU OE heterozygous male mice. Error bars are shown in curves (**A_1_**–**C_1_**). In (**A**–**C**) n=9-11 per mouse genotype; in (**D**) n=5 per mouse genotype. Error bars, ± SD; *P<0.05; **P<0.01.

To investigate the functional role of pancreas-targeted CLU upregulation in metabolic stress conditions, we performed an intraperitoneal GLU tolerance test in TgI173, TgI178 mice *vs.* non-Tg littermates. It was found that pancreas-specific young CLU Tg male mice developed higher GLU levels during the respective tolerance experiment (Figure 4A); thus, CLU Tg males likely have impaired GLU tolerance. Consistently, an INS tolerance test in the same mice groups revealed that both young and aged CLU Tg male mice tend to develop higher (*vs*. non-Tg littermate mice) GLU levels (Figure 4B) indicating that they are less INS tolerant. During fasting, tissues avoid hypoglycemia through glycogenesis and glucogenolysis from inorganic molecules, e.g., pyruvate (PYR) and lactic acid. PYR administration and the organism’s glycemic reaction is thus a marker of normal liver function and its ability to produce GLU. By performing an intraperitoneal PYR tolerance test in pancreas specific CLU Tg mice we found that young or aged male mice accumulated higher GLU levels, suggesting that they are also less PYR tolerant (Figure 4C); despite similar noted responses in female mice in these assays these were not statistically significant. Given these findings we measured fasting GLU levels in male TgN102 and TgG106 (ubiquitous CLU OE) mice and we also observed a significant induction in serum GLU levels (Supplementary Figure 1) indicating that they are likely in a hyperglycemic state. Finally, in a streptozotocin (STZ)-mediated diabetes induction model, we observed that STZ treated TgI173, TgI178 Tg animals (pancreas specific CLU OE) had constantly higher GLU levels in relation to control animals (Figure 4D), indicating an exaggeration of the STZ-induced diabetic phenotype. In support, tolerance tests showed that STZ treated-CLU overexpressing Tg (TgI173, TgI178) mice tend to be less (*vs*. non-Tg treated littermates) GLU, INS and PYR tolerant (Supplementary Figure 2).

Gene expression studies in isolated pancreatic and liver tissues from STZ treated (or not) TgI173 and TgI178 Tg mice, showed in the pancreas a trend for increased (*vs*. STZ treated non-Tg littermate mice) expression levels of the antioxidant genes *nrf2, nqo1* and *txnrd1*, as well as of genes involved in fatty acid synthesis (*acaca, srebpc1, fas*) and metabolic regulation (*gsk3a, gsk3β, pdp2, pdk1, pklR, mmtorc1, akt1)* (Supplementary Figure 3). In the liver, a trend for increased gene expression levels in STZ-treated CLU overexpressing mice (*vs*. non-Tg littermates) was observed for *nrf2;* for mitochondrial and mitostatic genes (*atp5a, ppargc1b, sdhA, pprc1, timm17b*), as well as for the metabolic genes *gsk3a, gsk3β, foxo1, foxo3, pdk1, akt1, gys1, gys2, g6pc* and *pepck* (Supplementary Figure 3). Thus, pancreas-targeted CLU OE causes metabolic deregulation being evident by altered expression of mitochondrial and metabolic genes, along with exaggeration of diabetic phenotypes as manifested by decreased GLU, INS and PYR tolerance in basal conditions or in a model of STZ-induced diabetes.

### Ubiquitous CLU OE alters proteostatic modules and mitigates cancer progression in a melanoma mouse tumor model

Given that ubiquitous CLU OE in mice tended to increase *nrf2* expression levels in the heart and muscle of Tg animals (see above), we investigated the possible interaction between CLU and proteostasis network modules. To this end, mouse embryonic fibroblasts (MEFs) were isolated from TgN102 and TgG106 lines and non-Tg littermate control animals. MEFs derived from Tg mice expressed higher levels of *clu* mRNA *vs*. controls (Supplementary Figure 4A); they also possessed higher (*vs*. controls) cathepsins B, L activity (Supplementary Figure 4B), while proteasome activity was higher in MEFs from the TgG106 line (Supplementary Figure 4C). Thus, increased CLU levels mobilize proteostatic modules.

**Figure 5:**
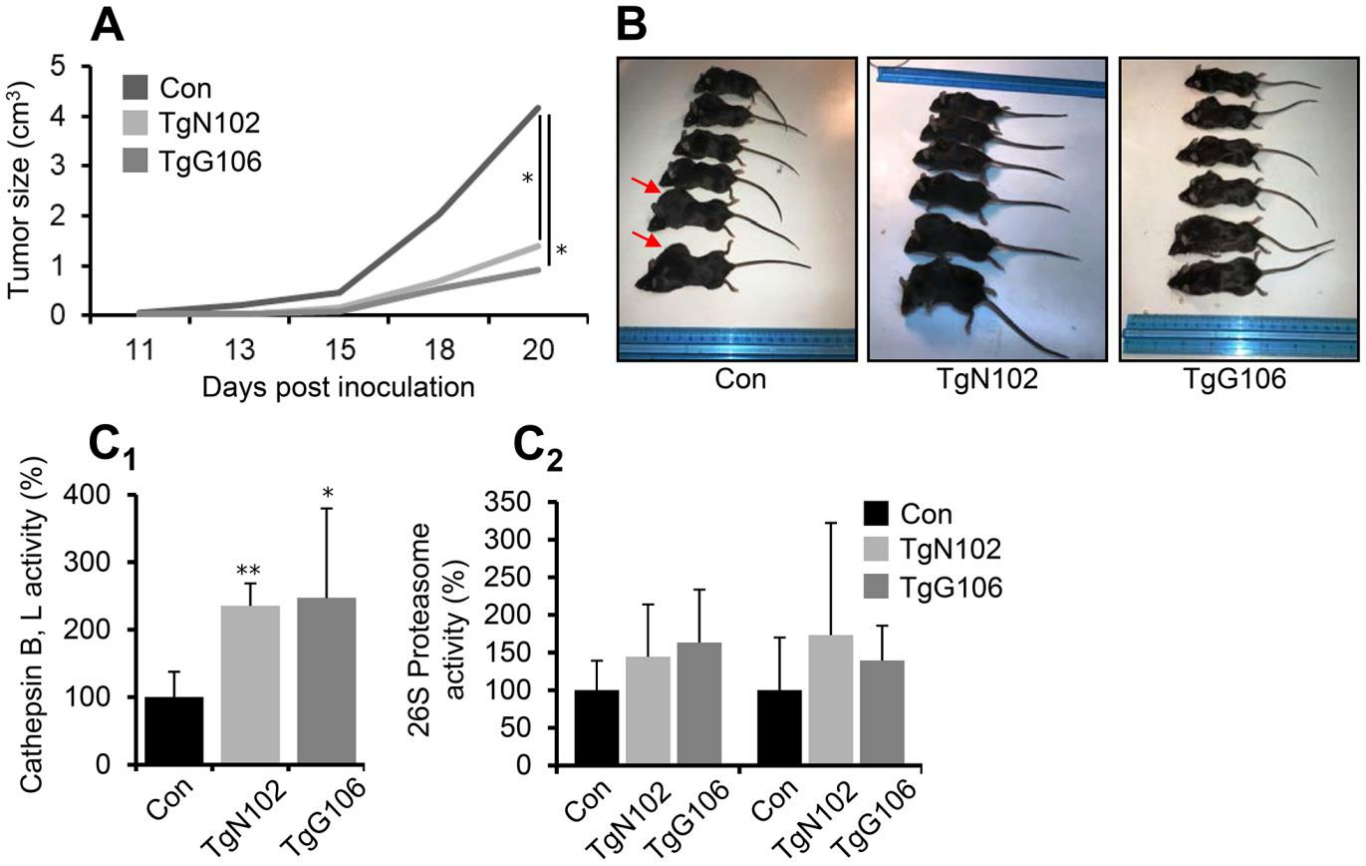
**Melanoma tumor cells growth is reduced *in vivo* at CLU Tg (ubiquitous OE) mice.** (**A**) Average tumor volume development by day 20, in control (Con; littermate non-Tg animals), TgN102 and TgG106 (ubiquitous CLU OE) mice after syngeneic melanoma tumor cells (B16.F1) inoculation. (**B**) Representative photos of control animals and of TgN102, TgG106 mice at the day of sacrifice. (**C**) Relative (%) cathepsins B, L (**C_1_**) and proteasome (**C_2_**) enzymatic activities in excised tumors of control and CLU overexpressing mice. Error bars, ± SD (n=6 per mouse genotype); *P<0.05; * *P < 0.01. Shown differences in (**A**) are also significant at days 13, 15 and 18 (P<0.05).

Since it was hypothesized that high CLU expression levels may suppress tumor progression at early, but not late, stages of carcinogenesis [1, 4, 10], we then investigated the functional implication of CLU OE in cancer. We developed a syngeneic mouse melanoma tumor model by grafting B16.F1 melanoma cells in the flank of control and CLU OE Tg (TgN102 and TgG106) mice. We found that tumors in non-Tg littermate C57Bl/6 mice become pulpable earlier and grew significantly faster as compared to tumors developed in CLU Tg animals (Figure 5A, 5B). CLU Tg mice were also characterized by enhanced lysosomal cathepsins B, L (Figure 5C_1_) and a trend for increased (not significant) proteasomal (Figure 5C_2_) enzymatic activities further verifying proteostatic modules activation in CLU TgN102 and TgG106 lines. Further studies revealed a significant downregulation (*vs*. littermate non-Tg mice) in a panel of antioxidant, proteasome, autophagy-related, mitochondrial, and metabolic genes in tumors grown in CLU TgN102 and TgG106 mice (Supplementary Figure 5A); this readout was particularly enhanced in genes encoding enzymes that contribute to Warburg effect in cancer cells. Furthermore, grafting melanoma tumor cells in mice increased serum CLU levels in control but also in Tg mice (Supplementary Figure 5B) suggesting a possible role of circulating CLU in suppressing tumor promotion in CLU OE Tg mice. Overall, ubiquitous OE of CLU exerts a tumor suppressive role in the melanoma mouse tumor model.

## DISCUSSION

CLU is an exciting chaperone whose different isoforms likely function both intra- and extra-cellularly [11]. Here, we report the establishment of CLU overexpressing Tg mice showing that ubiquitous CLU upregulation modulates antioxidant, proteostatic and metabolic genes. CLU was characterized as a sensitive cellular biosensor of oxidants that functions to protect cells from the deleterious effects of oxidative stress [12]; also, it was found to stabilize the Ku70-Bax complex, preventing Bax protein from activating the mitochondrial apoptotic pathway [11] and to cause juxtanuclear aggregate formation and mitochondrial alteration [13].

Targeted CLU OE in the pancreas was induced based on previous studies that indicated a close link between CLU expression levels and diabetes [8, 14]. Indeed, pancreas-targeted CLU OE also induced higher CLU expression levels in the liver, modulated metabolic genes and impaired GLU, INS and PYR tolerance of Tg mice. These traits are usually accompanied with reduced GLU uptake into the INS-sensitive tissues (e.g., skeletal muscle, liver, and adipose tissue) and are signs of metabolic syndrome/INS resistance that can lead to diabetes and atherosclerosis [15]. Consistently, previous findings have showed that elevated plasma CLU levels are associated with INS resistance markers [9]. Yet, given the different *clu* transcripts that have been identified in physiological or stress conditions [16–20] along with the fact that the exact structure of the mature protein is not known [20], the functional role of CLU in human pathologies, including metabolic syndrome, remains to be clarified. In a STZ-mediated diabetes model, pancreas-targeted CLU OE induced significantly higher GLU levels, as well as GLU, INS and PYR decreased tolerance indicating a more severe diabetic phenotype in those Tg animals. It has been shown before that increased serum CLU levels are linked with T2D [8, 13] and that high fat diet in CLU deficient mice led to increased INS resistance [21]; in this model it was proposed that CLU protects from INS resistance by reducing oxidative stress. Since we found that increased CLU levels in pancreas and liver from the tissue specific CLU Tg mice do not induce premature onset of diabetes but exacerbate INS intolerance, we suggest that pancreas specific CLU upregulation may result in deregulation of the GLU-INS metabolic pathway. In support, CLU was identified recently as a hepatokine that targets muscle (a tissue particularly enhanced in males) GLU metabolism and INS sensitivity through low-density lipoprotein receptor-related protein-2 along with the INS receptor signaling cascade [22]. Elevated GLU levels upregulate the metabolic genes *akt*, *foxo1*, *pgc-1*, *g6pc* and *pepck* in the liver, where activation of the phosphoinositol 3-kinase/AKT pathway inhibits the rate-controlling enzymes of gluconeogenesis and promotes glycogen synthesis [23, 24]. In STZ-treated pancreas CLU overexpressing Tg mice, activation of the AKT pathway might be a countereffect aiming to increase GLU uptake in the tissues. Nevertheless, upon diabetes induction, where pancreatic β-cells are destroyed and no INS is produced, CLU OE seems to promote *foxo1* activation, which along with its co-activator *pgc-1* induce transcription of *g6pc* and *pepck* enzymes that participate in GLU production [25] in the liver; a fact that explains the increased GLU levels observed. Probably, the observed hyperglycemia can be also attributed to impaired uptake of GLU from skeletal muscle and liver and in general all INS-dependent tissues. Elevated expression levels of the transcription factor *Srebp1c* and its targets *scd-1* and *acl* are also observed in conditions of excessive GLU production, to promote lipogenesis in the liver [26] and a free fatty acid flux into the liver which also contributes to hepatic INS resistance [27]. Our results show that *Srebp1c* levels drop in both groups after STZ administration; yet, in STZ-treated pancreas-CLU overexpressing mice its levels remain higher than in control mice possible due to increased fatty acid oxidation. It has been stated before that during prolonged INS resistance, *Srebp1c* levels increase, to initiate *de novo* fatty acid biosynthesis [28]; however, CLU OE in hepatocytes downregulated *Srebp1c* expression [29]. In support to the proposed mitochondrial-metabolic deregulation in our CLU OE Tg models, chronic diabetes induced by STZ provoked significant alterations in hepatic mitochondrial function [30] and STZ-induced cytotoxicity in HepG2 cells is also mediated by oxidative stress and mitochondrial dysfunction [31]; moreover, mitochondria function was compromised in diabetic and prediabetic humans [32, 33]. Induction of diabetes also leads to decreased expression levels of the transcription factor FOXO6. Elevated FOXO6 levels in the liver led to gluconeogenesis and increased GLU levels during fastening, that were downregulated by INS mediated FOXO6 suppression through phosphorylation and inactivation of its transcriptional activity [34]. Our data suggest that CLU likely interacts with FOXO6 in the liver to reduce GLU levels. Pancreatic β-cells elimination due to STZ administration indicate that CLU in our Tg animals could be produced either from the remaining β-cells; from enhanced β-cells regeneration [35] or from the liver. The mechanistic details behind these observations should however await further future studies.

Furthermore, we observed that ubiquitous CLU OE delays the growth of melanoma tumor cells being grafted in Tg mice. CLU action during carcinogenesis is a continuous field of study since CLU has been implicated in tumor cells survival, epithelial–mesenchymal transition, metastasis and chemoresistance [4, 11, 15, 36]. CLU seems to promote cancer at more advanced stages of the disease, while at early stages, in agreement with our ubiquitous CLU overexpressing *in vivo* model, it likely exerts a suppressive role [37–40]. We propose that increased circulating or intracellular CLU levels may, via its chaperone activity, establish a tumor suppressive micro-environment by inhibiting tumor promoting proteotoxic stress. Indeed, important key enzymes involved in the Warburg effect like hexokinase 4 (HEX4), pyruvate kinase muscle isozyme 2 (PKM2) and lactate dehydrogenase (LDHA) are downregulated in CLU overexpressing mice, while given that HEX2 and PKM2 are substrates of chaperone mediated autophagy [41, 42], CLU OE may, as reported before [43–45], also modulate autophagic responses. Moreover, in the grafted tumors of CLU OE mice c-MYC and its targets, e.g., glucose transporters (GLUT1-4), LDHA and PKM2 [46] are downregulated. Hypoxia-inducible factor 1 alpha (HIF1α) reduction is also followed by reduced expression of glycolytic enzymes like hexokinase II (HEX2) and pyruvate dehydrogenase kinase 1 (PDK1), an inhibitor of the tricarboxylic acid cycle [47]. Finally, CLU OE was found to (among others) decrease PGC1a levels, which reportedly promotes metastasis by mediating mitochondrial biogenesis [48].

Taken together, our observations provide *in vivo* evidence which corroborate the notion that CLU is a potential modulator of metabolic and/or proteostatic pathways playing a significant functional role in diabetes and tumorigenesis.

## MATERIALS AND METHODS

### Use of animals

Mice were maintained under specific pathogen-free conditions in the facilities of the Department of Animal Models for Biomedical Research of the Hellenic Pasteur Institute (Facilities License Numbers: ELBIO11, ELBIO12 and ELBIO13). Animals were housed at room temperature 22 ± 2° C, relative humidity 40-70% and 12 hours light/12 hours dark cycle. All mice procedures were assessed by the Institutional Protocol Evaluation Committee and licenses were issued by national authorities, according to the Greek Law 56/2013, in conformity with European Union guidelines; PD 56/2013 and European Directive 2010/63/EU, welfare and ethical use of laboratory animals based on 3+1R. The experimental protocols have been positively evaluated by the Institutional Protocol Evaluation Committee and were licensed under the registered codes 987/10.02.2012 and 2582/29-05-2018, by the Official Veterinary Authorities of Attika (Greece) Prefecture.

### Generation of CLU overexpressing mice

To establish Tg mice overexpressing CLU ubiquitously, mouse CLU cDNA was inserted in a hβ*actin* promoter cassette. The plasmid was microinjected into pronuclei of F1 (CBA/CaOla × C57BL/6 OlaHsd)-fertilized oocytes, as described previously [49]. Two Tg lines, Tg.*hβactin.clu*, were produced (TgN102, TgG106) that transmitted the transgene in a Mendelian way. To obtain pancreas-targeted CLU overexpressing mice, the mouse CLU cDNA was inserted in a pancreatic and duodenal homeobox 1 (*pdx-1*) gene promoter cassette. The plasmid was microinjected into pronuclei of F1 (CBA/CaOla × C57BL/6 OlaHsd)-fertilized oocytes, as above. Two Tg lines, Tg.*pdx-1.clu*, were produced (TgI173, TgI178) that transmitted the transgene in a Mendelian way. All generated Tg mice lines were backcrossed to the C57BL/6 background for at least 10 generations and are registered in the resources of HPI as (B6-Tg(h*βactin.clu*)N102HP and G106HP, as well as B6-Tg(*pdx-1.clu*)I173HP and I178HP. To identify Tg mice, genomic DNA was amplified with primers specific for *CLU* cDNA: forward, 5′- GAT CTT GTC TGT GGA CTG TTC A-3′, and reverse, 5′- CTA TCT CAT TCC GCA CGG CTT-3′. All mice showed no pathological phenotypic characteristics and they reproduced normally.

### CLU deficient mice

CLU-deficient mice (CLU KO) backcrossed to the C57Bl/6 strain for more than 10 generations were obtained at the Animal Facility of the University of Parma by breeding heterozygous parents. Mice were housed in a standard animal facility under controlled environmental conditions (22 ± 2° C, 12 hours light/dark cycle) and were allowed free access to food and water. Genotyping of the offspring was performed by PCR amplification of DNA extracted from ear biopsies as described before [50]. A total of 8 mice (2 male CLU KO, 2 male WT, 2 female CLU KO and 2 female WT), aged 7-8 months underwent blood withdraw by retro-orbital bleeding. Then, animals were sacrificed by cervical dislocation, tissues were collected and quick frozen in liquid nitrogen and stored at -80° C until use. All experimental procedures involving CLU KO mice were approved and conducted in accordance with the Italian law (D.lgs 26/2014).

### Preparation of tissue protein extracts, SDS-PAGE and immunoblot analysis

Tissue and tumor extracts from experimental and control (littermate non-Tg mice) mice were lysed with NP-40 lysis buffer containing protease and phosphatase inhibitors (Sigma-Aldrich, USA). Protein content of samples was assessed by Bradford (Bio-Rad Laboratories, UK). SDS-PAGE and immunoblotting assays were performed, as described previously [51]. Primary and horseradish peroxidase-conjugated (Jackson Laboratories) secondary antibodies were applied for 1 h at room temperature (RT) and were developed by using an enhanced chemiluminescence reagent kit (Bio-Rad Laboratories). Primary antibodies used were against CLU (Santa Cruz, SC-6419) and GAPDH (Sigma, G9545).

### Isolation of MEFs, Real-Time PCR and measurement of proteasome, cathepsins B, L activities

Isolation of MEFs was done as described previously [52]. RNA extraction from mouse tissue or tumor extracts, cDNA synthesis and Real-Time PCR, along with measurement of proteasome and cathepsins B, L activities in cells, tissues or tumor extracts was done as described previously [53, 54]; for details see Supplementary Materials and Methods.

### Intraperitoneal GLU, INS and PYR tolerance tests; GLU, INS measurements in mice plasma

Control (littermate non-Tg mice) or experimental mice (see Figure legends) were fasted overnight. Blood samples were collected from the tail vein prior to intraperitoneal injection of GLU (1 g/kg, Sigma-Aldrich), INS (1 mU/g bodyweight, Pharmaserve, Greece) or sodium PYR (2 g/kg, Applichem), respectively. Blood samples were collected at 15-, 30-, 60-, and 120-min post-injection of substances [55]. GLU and INS levels in isolated murine plasma were measured in an external veterinary diagnostic lab.

### Streptozotocin inducible diabetes model

Male mice overexpressing CLU in pancreas and littermate non-Tg mice (control) were injected with streptozotocin (STZ, Sigma-Aldrich) for 5 consecutive days. STZ was dissolved in 0.1 M sodium citrate buffer (pH 4.5) and was injected intraperitoneally (40 mg/kg) within 15 min of dissolution; the control group received citrate buffer solution.

### Syngeneic melanoma inducible tumor model

Mice ubiquitously overexpressing CLU (~25 g of weight, 6-8 weeks of age) and littermate non-Tg mice (control) were subcutaneously inoculated with 10^5^ B16.F1 melanoma cells. Tumor growth rate was recorded every 2 days by measuring the major and minor axes of the formed tumors with a digital caliper. Measurements were transformed into tumor volume using the formula: tumor volume (cm^3^) = major axis x minor axis^2^ x 0.5. On day 22, animals were euthanized by cervical dislocation and tumors were excised for RNA extraction, immunoblotting, proteasome and cathepsins B, L activity measurements.

### Statistical analysis

All experiments were performed in triplicates and data were statistically analyzed with the use of ANOVA single factor. Level of correlation among different analyzed groups was calculated by the Pearson correlation coefficient, r.

### Data availability

The datasets generated and/or analyzed during the current study are available from the corresponding author on reasonable request.

## Supplementary Material

Supplementary Materials and Methods

Supplementary Figures

Supplementary Tables
