# Supplementary material for: Clusterin overexpression in mice exacerbates diabetic phenotypes but suppresses tumor progression in a mouse melanoma model
Source: Aging (Albany NY). 2021 Mar 10;13(5):6485–505. doi: 10.18632/aging.202788 (PMC7993736; doi:10.18632/aging.202788)
Supplement: Supplementary Materials and Methods [file aging-13-202788-s001.pdf]

SUPPLEMENTARY MATERIALS

RNAlater (Sigma-Aldrich) and the RNeasy mini kit (Qiagen) were used for total RNA isolation from tissue or tumor samples according to the manufacturer's instructions. cDNA synthesis and Quantitative Real-time PCR were performed as described before (Cheimonidi et al., 2018). Primers were designed using the primer-BLAST tool (http://www.ncbi.nlm.nih.gov/tools/primer-blast/) and were the following: *b-ACTIN*-F: GGC-TGT-ATT-CCC-CTC-CAT-CG, *b-ACTIN*-R: CCA-GTT-GTT-AAC-AAT-GCCA-TGT; *CLU*-F: GCA-GGA-GGT-CTC-TGA-CAA-TGA, *CLU*-R: GAC-GGC-GTT-CTG-AAT-CTC-CT; *NRF2*-F: CCA-GGA-CTA-CAG-TCC-CAG-CAG, *NRF2*-R: CTC-CAA-GAT-CTA-TGT-CTT-GCC-TCC; *TXNRD1*-F: CCA-TCG-GTG-ACA-TCC-TGG-AG, *TXNRD1*-R: CTC-TGA-GCC-AGC-AAT-CTC-CC; *NQO1*-F: CAT-TGC-AGT-GGT-TTG-GGG-TG, *NQO1*-R: TCT-GGA-AAG-GAC-CGT-TGT-CG; *PSMA7*-F: ATC-AAC-AGA-GCC-CGG-GTA-GA, *PSMA7*-R: GCC-GAG-ATA-CCA-AAT-GGC-CT; *PSMB5*-F: AAT-GCT-TCA-CGG-AAC-CAC-CA, *PSMB5*-R: CTT-CAC-CGT-CTG-GGA-AGC-AA; *CATHEPSIN L*-F: AAT-GGA-GGT-CTG-GAC-TCG-GA, *CATHEPSIN L*-R: CAG-CGA-ACT-CGG-CTC-TGT-AT; *BECLIN1*-F: GGA-AGT-AGC-TGA-AGA-CCG-GG, *BECLIN1*-R: TTA-GAC-CCC-TCC-ATG-CCT-CA; *LC3B*-F: GCT-CGC-TGC-TGT-CTA-GAT-GT, *LC3B*-R: CAG-TCG-CTT-AAG-CTG-GGT-CA; *HDAC6*-F: TCA-GCC-TCA-ACT-GGT-CTT-GG, *HDAC6*-R: AGC-AAA-TGG-GTT-AGG-TGG-GC; *P62*-F: CTT-CGG-AAG-CTG-AAA-CAT-GGA-C, *P62*-R: TGA-CAT-TGG-GAT-CTT-CTG-GTG-G; *GSK3a*-F: CAG-AGA-CGA-GGG-AAC-TGG-TG, *GSK3a*-R: CAG-TGG-TCC-AGC-TTA-CGC-A; *GSK3b*-F: TAG-TCG-AGC-CAA-GCA-GAC-AC, *GSK3b*-R: TGT-CTC-GAT-GGC-AGA-TTC-CAA; *PDK1*-F: ACG-GGA-CAG-ATG-CGG-TTA-TC, *PDK1*-R: GCT-TCC-AGG-CGG-CTT-TAT-TG; *PDP2*-F: AGG-AGA-GGA-CGA-GGA-TAC-GAG, *PDP2*-R: CTC-CCA-CCT-CGT-AAA-AGA-GCA; *PKLR*-F: GGC-AGA-TGA-TGT-GGA-CCG-AA, *PKLR*-R: CCA-GAT-CAC-CAA-CTC-GGA-GG; *FOXO3*-F: GGT-ACC-AGG-CTG-AAG-GAT-CA, *FOXO3*-R: CGT-GGG-AGT-CTC-AAA-GGT-GT; *FOXO1*-F: TCA-AGG-ATA-AGG-GCG-ACA-GC, *FOXO1*-R: CCT-CCC-TCT-GGA-TTG-AGC-ATC; *PEPCK*-F: AAG-AAG-AAA-TAC-CTG-GCC-GCA, *PEPCK*-R: TTT-GTC-TTC-ACT-GAG-GTG-CCA; *AKT1*-F: CCA-AGG-AGA-TCA-TGC-AGC-AC, *AKT1*-R: TAC-CTG-GTG-TCA-GTC-TCA-GAG-G; *MTOR*-F: CCA-TCA-ATC-TGA-TGC-TGG-A, *MTOR*-R: GGTGT-GGC-ATG-TGG-TTC-TGT; *INSR*-F: TGG-CAT-GGC-ATA-CTT-GAA-CG, *INSR*-R: TTG-CCC-CCT-TTC-CGA-TAG-TA; *GYS-1*-F: CAC-AGA-ACG-GTTGTC-GGA-CT, *GYS-1*-R: GTG-AAG-TGG-TCT-GGA-AAG-GC; *GYS-2*-F: TAA-ACA-GTC-ACG-CCG-GCA-AA, *GYS-2*-R: TTG-TCT-GGA-AAA-GCC-CTG-CT; *PKM2*-F: TGC-AAT-TAT-TTG-AGG-AAC-TCC, *PKM2*-R: CAC-TGC-AGC-ACT-TGA-AGG-AG; *GLUT1*-F: CAT-CCT-TAT-TGC-CCA-GGT-GTT-T, *GLUT1*-R: GAA-GAC-GAC-ACT-GAG-CAG-CAG-A; *GLUT4*-F: AAA-AGT-GCC-TGA-AAC-CAG-AG, *GLUT4*-R: TCA-CCT-CCT-GCT-CTA-AAA-GG; *HK2*-F: TGA-TCG-CCT-GCT-TAT-TCA-CGG, *HK2*-R: AAC-CGC-CTA-GAA-ATC-TCC-AGA; *FH*-F: GAG-AGC-TGA-TCT-TGC-CTG-AA, *FH*-R: ACA-CTG-AGT-AGG-GTT-CAC-CT; *LDHA*-F: TGT-CTC-CAG-CAA-AGA-CTA-CTG-T, *LDHA*-R: GAC-TGT-ACT-TGA-CAA-TGT-TGG-GA; *SDHA*-F: TGT-GCG-CAC-TGC-AGA-CCA-TA, *SDHA*-R: CAA-ACG-GCT-TCT-TCT-GCT-GTC; *ATP5B*-F: ATG-CAG-GAA-AGG-ATC-ACC-ACC, *ATP5B*-R: AGC-AAT-AGC-CCG-GGA-CAA-C; *FAS*-F: GCT-GCG-GAA-ACT-TCA-GGA-AAT, *FAS*-R: AGA-GAC-GTG-TCA-CTC-CTG-GAC-TT; *SCD-1*-F: CTG-ACC-TGA-AAG-CCG-AGA-AG, *SCD-1*-R: GCG-TTG-AGC-ACC-AGA-GTG-TA; *ACL*-F: GCC-AGC-GGG-AGC-ACA-TC, *ACL*-R: CTT-TGC-AGG-TGC-CAC-TTC-ATC; *ACAC*-F: GCC-TCT-TCC-TGA-CAA-ACG-AG, *ACAC*-R: TGA-CTG-CCG-AAA-CAT-CTC-TG; *FOXO6*-F: AGA-GCG-CCC-CGG-ACA-AG-AGA, *FOXO6*-R: GCC-GAA-TGG-AGT-TCT-TCC-AGC-C; *SREBPC*-1-F: CCA-TCG-ACT-ACA-TCC-GCT-TCT-T, *SREBPC*-1-R: ACT-TCG-CAG-GGT-CAG-GTT-CTC; *APOE*-F: ACA-GAT-CAG-CTC-GAG-TGG-CAA-A, *APOE*-R: ATC-TTG-CGC-AGG-TGT-GTG-GAG-A; *TIMM17A*-F: ATT-GAA-GGA-GCT-GGT-ATC-TTG-C, *TIMM17A*-R: CGG-TAG-TCT-CCA-AAC-GGC-G; *TIMM17B*-F: CAG-GCT-ATC-AAG-GGC-TTC-CG, *TIMM17B*-R: TCC-TCA-CAG-CAT-TGA-CAC-TAC-C; *TFAM*-F: CAG-GAG-GCA-AAG-GAT-GAT-TC, *TFAM*-R: CCA-AGA-CTT-CAT-TTC-ATT-GTC-G; *PGC1a*-F: GTA-AAT-CTG-CGG-GAT-GAT-GG, *PGC1a*-R: AGC-AGG-GTC-AAA-ATC-GTC-TG; *PPARGC1b*-F: GGA-GAC-ACA-GAT-GAA-GAT-CCA-AGC, *PPARGC1b*-R: GCT-CCA-CCG-TCA-GGG-ACT-C; *PPRC1*-F: CAG-GAG-AAG-AAG-CCC-TTA-GAC-C, *PPRC1*-R: CTT-TCG-CCA-AGA-GTG-AGA-CAG; *MPC1*-F: AAC-TAC-GAG-ATG-AGT-AAG-CGG-C, *MPC1*-R: GTG-TTT-TCC-CTT-CAG-CAC-GAC; *BAX*-F: TAG-CAA-ACT-GGT-GCT-CAA-GG, *BAX*-R: TCT-TGG-ATC-CAG-ACA-AGC-AG; *KU70*-F: CCC-AAG-GTT-GAA-GCC-ATA-AA, *KU70*-R: TTA-CGA-AAA-TGG-GCC-TTC-AG; *P53*-F: GTA-TTT-CAC-CCT-CAA-GAT-CC, *P53*-R: TGG-GCA-TCC-TTT-AAC-TCT-A; *HIF1A*-F: TCA-AGT-CAG-CAA-CGT-GGA-AG, *HIF1A*-R: TAT-CGA-GGC-TGT-GTC-GAC-TG; *MYC*-F: TGA-GCC-CCT-AGT-GCT-GCA-T, *MYC*-R: AGC-CCG-ACT-CCG-ACC-TCT-T.

Measurement of proteasome and cathepsin B, L activities in cells or tumor extracts

Isolated cells or tumor samples were lysed in the presence of protease and phosphatase inhibitors (Sigma-Aldrich); protein content of samples was assessed with Bradford (Bio-Rad Laboratories). Proteasome and cathepsin B, L activities were measured as described before (Cheimonidi et al., 2018).
