# Supplementary material for: Clusterin overexpression in mice exacerbates diabetic phenotypes but suppresses tumor progression in a mouse melanoma model
Source: Aging (Albany NY). 2021 Mar 10;13(5):6485–505. doi: 10.18632/aging.202788 (PMC7993736; doi:10.18632/aging.202788)
Supplement: Supplementary Figures [file aging-13-202788-s002.pdf]

SUPPLEMENTARY FIGURES

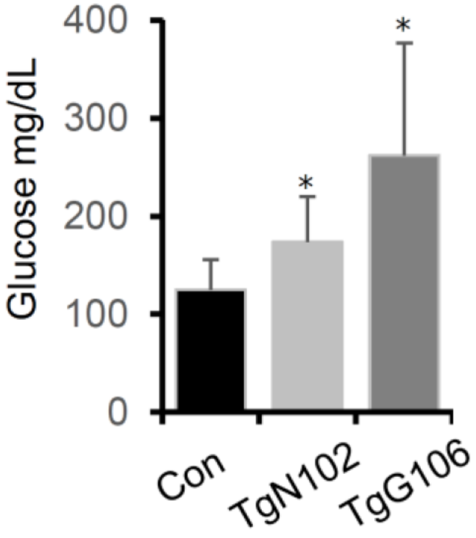

Supplementary Figure 1. Fasting GLU levels in control and CLU Tg (ubiquitous OE) male mice. GLU levels in control (littermate non-Tg) and CLU Tg male mice following fasting (n=4 per mouse genotype). Error bars, ± SD; *P<0.05.

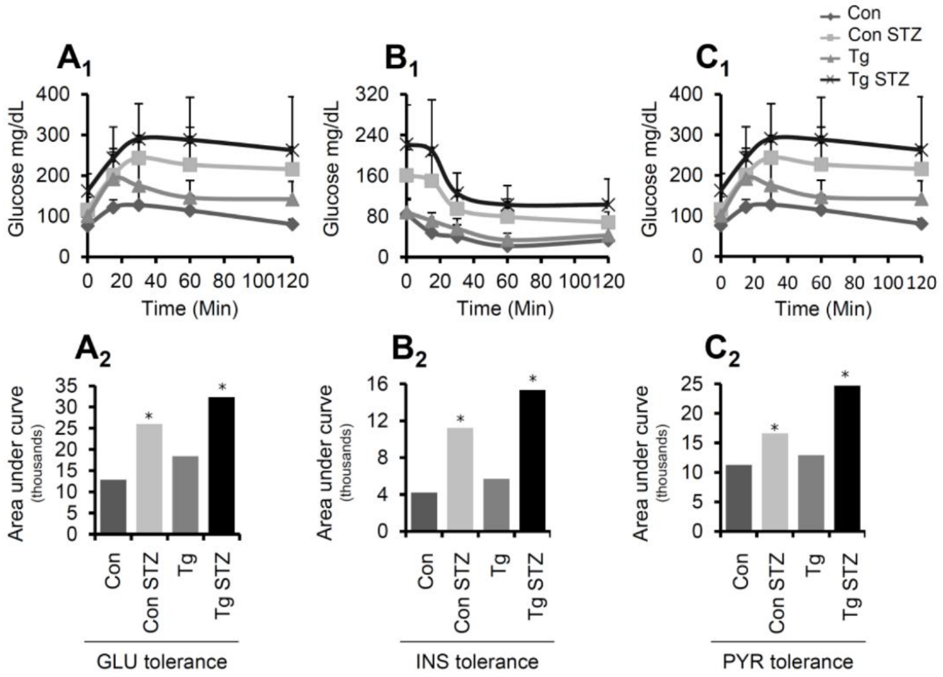

Supplementary Figure 2. GLU, INS and PYR decreased tolerance of CLU Tg (pancreas targeted OE) mice, is exacerbated in a model of STZ-mediated induction of diabetes. GLU levels (tolerance curves) in control (Con; littermate non-Tg) mice, control mice being treated with STZ (Con STZ), CLU overexpressing (Tg; pancreas-targeted) mice and CLU overexpressing mice being treated with STZ (Tg STZ). Mice were administered GLU (**A_1**), INS (**B_1**) or PYR (**C_1**); GLU levels were measured before GLU, INS, PYR injection (see, Materials and Methods) and 15, 30, 60 and 120 minutes after. (**A_2–C_2**) Areas under respective curves (**A_1–C_1**) being calculated from the sum of the different trapeziums formed (errors bars are shown in curves). (n=5 per mouse genotype). Error bars, ± SD; **P*<0.05.

PANCREAS

Gene	Con STZ *vs.* Con	Tg STZ vs. Tg
CLU	1.50	1.67
GSK3A	0.97	1.24
GSK3B	1.44	3.11
FOXO1	2.09	1.55
FOXO3	2.04	3.03
PDP2	0.77	2.06 *
PDK1	0.91	1.70
AKT1	1.30	2.55
PKLR	0.45	4.34
G6PC	6.12	14.60
PEPCK	1.70	1.42
GYS1	2.00	1.55
GYS2	0.74	33.75
INSR	1.41	0.63
APOE	0.36	1.26
APOA1	0.71	61.05
FOXO6	1.09	1.86
ACACA	0.24	1.10 *
SCD1	1.53	0.34
ACL	1.19	1.17
SREBPC1	0.59	1.46
MMTORC1	0.37	1.27
FAS	0.63	1.01
ATP5A	0.96	1.46
PGC1A	2.82	3.31
PPARGC1B	1.28	1.24
SDHA	0.86	1.27
PPRC1	3.16	1.58
TFAM	0.69	0.89
TIMM17A	0.34	1.48
TIMM17B	1.22	1.12
NRF2	0.57	1.64
NQO1	0.07	1.81
TXNRD1	0.56	1.80

Pearson Corr. 0.040

LIVER

Gene	Con STZ *vs.* Con	Tg STZ vs. Tg
CLU	0.36	1.86
GSK3A	0.08	0.64
GSK3B	0.04	1.40
FOXO1	0.46	2.66 **
FOXO3	0.76	3.34 *
PDP2	0.98	0.71
PDK1	0.35	1.13 *
AKT1	0.94	12.44 *
PKLR	0.58	0.85
G6PC	0.39	2.83
PEPCK	0.49	2.88 *
GYS1	1.00	2.45 *
GYS2	1.05	3.00
INSR	0.64	1.09
APOE	1.16	0.24
APOA1	0.13	3.19
FOXO6	0.91	0.43
ACACA	0.82	0.56
SCD1	0.54	0.93 *
ACL	0.50	1.52 **
SREBPC1	0.34	0.77 **
MMTORC1	1.46	0.55
FAS	0.97	0.75
ATP5A	0.37	1.97 *
PGC1A	1.31	2.75
PPARGC1B	0.33	1.55 **
SDHA	0.43	1.25 *
PPRC1	0.18	1.93 **
TFAM	1.05	0.66
TIMM17A	1.01	1.42 **
TIMM17B	0.45	1.12 *
NRF2	0.31	1.11 **
NQO1	0.87	0.62
TXNRD1	0.60	0.58

Pearson Corr. 0.057

Lowest value — 1 — Highest value

Fold change
* P<0.05 Tg STZ *vs.* Con STZ treatment
**P<0.01 Tg STZ *vs.* Con STZ treatment

Supplementary Figure 3. Expression levels (heat map) of antioxidant, mitochondrial and metabolic genes in the pancreas and liver of control or CLU Tg (pancreas-targeted OE) mice (*vs*. non STZ treated animals) after STZ administration-mediated induction of diabetes. Heat map indicating relative expression levels of shown genes following STZ treatment in control (Con; littermate non-Tg) or TgI173, TgI178 mice; differential responses among the different animal groups are also evident by shown Pearson Correlation (*r*) values (Con *vs*. Tg columns). The different groups of animals tested are control (Con; littermate non-Tg) mice, control mice being administered STZ (Con STZ), CLU overexpressing mice (Tg; pancreas-targeted) and CLU overexpressing mice being administered STZ (Tg STZ) (n=5 per mouse genotype). *P<0.05; **P<0.01 (Tg STZ *vs*. Con STZ treatment).

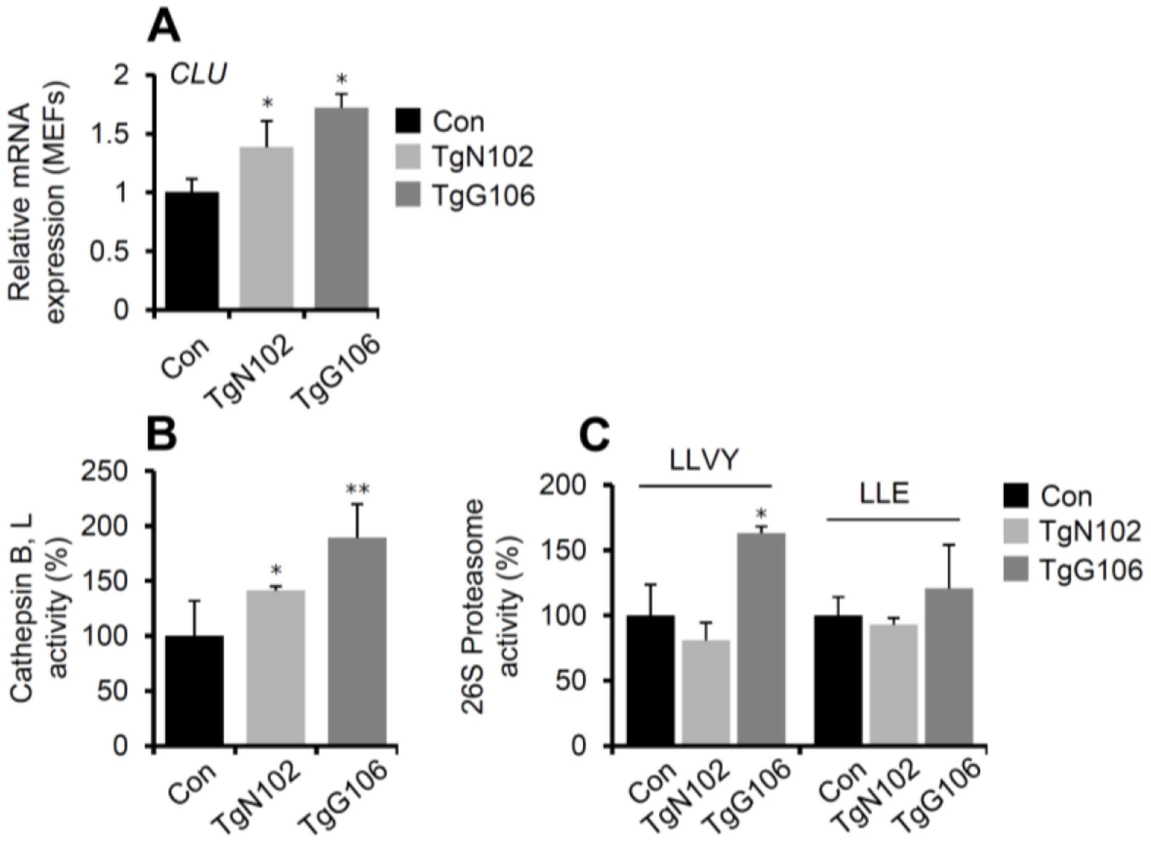

Supplementary Figure 4. CLU OE upregulates proteostatic modules in MEFs derived from TgN102 or TgG106 (ubiquitous OE) mice. (**A**) Relative *clu* mRNA expression levels in control (Con; littermate non-Tg), TgN102 and TgG106 mice derived MEFs. (**B**) Relative (%) cathepsin B, L enzymatic activities in control, TgN102 and TgG106 MEFs. (**C**) Relative (%) proteasome enzymatic activities in control, TgN102 and TgG106 MEFs. Error bars, ± SD (n=2-4 per mouse genotype); *P<0.05; **P<0.01.

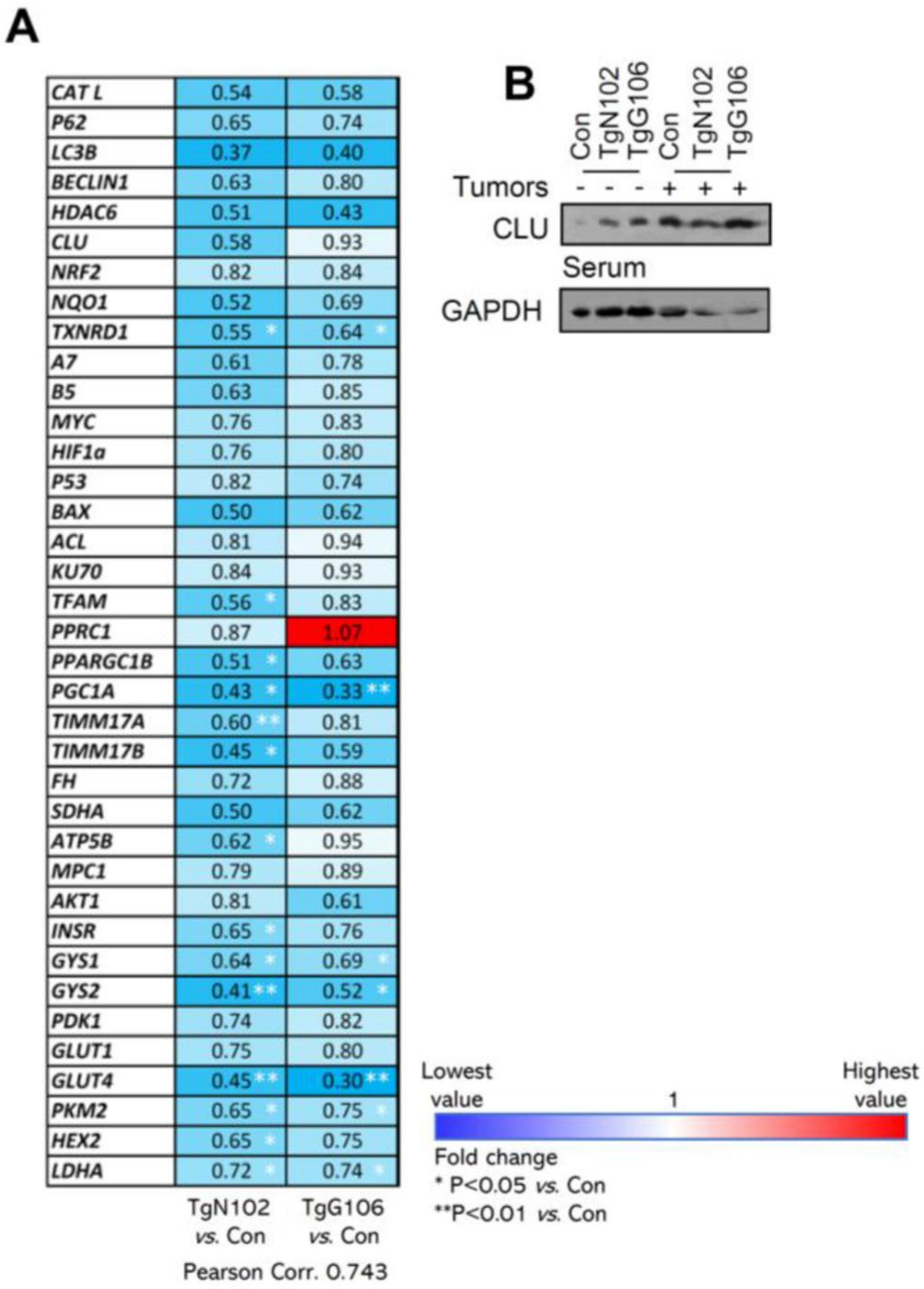

Supplementary Figure 5. Differential (*vs*. controls) gene expression levels (along with CLU serum levels in Tg mice) in melanoma tumors grafted in TgN102 and TgG106 (ubiquitous OE) mice. (**A**) Heat map indicating relative expression levels (*vs*. Con; littermate non-Tg) of shown autophagic, proteasome, antioxidant, mitochondrial and metabolic genes in melanoma cells-derived tumors grafted in TgN102 or TgG106 mice (n=6 per mouse genotype); similar genomic responses in Tg mice are also evident by shown Pearson Correlation (*r*) values (TgN102 *vs*. TgG106). (**B**) CLU protein levels in the serum of shown mice bearing (or not) melanoma cells-derived tumors (n=6 per mouse genotype). GAPDH probing (tissue lysate) was used as a reference. In (A), **P*<0.05; ***P*<0.01 (Tg *vs*. Con).
