# Supplementary material for: Clusterin overexpression in mice exacerbates diabetic phenotypes but suppresses tumor progression in a mouse melanoma model
Source: Aging (Albany NY). 2021 Mar 10;13(5):6485–505. doi: 10.18632/aging.202788 (PMC7993736; doi:10.18632/aging.202788)
Supplement: Supplementary Tables [file aging-13-202788-s003.pdf]

SUPPLEMENTARY TABLES

Supplementary Table 1. Statistical analyses (Figure 2A).

	BRAIN		LIVER		INTESTINE		HEART		MUSCLE	
	TgN102	TgG106	TgN102	TgG106	TgN102	TgG106	TgN102	TgG106	TgN102	TgG106
Pearson Corr.[1]		0.521		0.619		0.758		0.349		0.542
Pearson Corr.[2]				-0.043 Brain		0.186 Liver		-0.289 Intestine		-0.244 Heart
						0.265 Brain		-0.097 Liver		0.086 Intestine
								-0.258 Brain		0.558 Liver
										0.151 Brain
Regression		0.055		0.010		0.001		0.184		0.045
(*F* significance)				0.826 Brain		0.323 Liver		0.120 Intestine		0.210 Heart
						0.172 Brain		0.596 Liver		0.673 Intestine
								0.184 Brain		0.002 Liver
										0.479 Brain

Pearson Corr.[1] denotes gene expression correlation within tissue amongst the two Tg lines, and Pearson Corr.[2] across tissues (n=4 per mouse genotype).
Regression analysis (*F* significance) refers to genes studied in either Tg lines or tissues.

Supplementary Table 2. Statistical analyses (Figure 2B).

	LIVER	INTESTINE	HEART	MUSCLE
	CLU KO	*CLU* KO	*CLU* KO	*CLU* KO
Pearson Corr.[2]		-0.239 Liver	0.470 Intestine	-0.431 Heart
			-0.086 Liver	-0.070 Intestine
				0.337 Liver
Regression (*F* significance)		0.370 Liver	0.066 Intestine	0.094 Heart
			0.749 Liver	0.795 Intestine
				0.200 Liver

Pearson Corr.[2] across tissues (n=4 per mouse genotype).
Regression analysis (*F* significance) refers to genes studied in either Tg lines or tissues.

Supplementary Table 3. Statistical analyses (Figure 3C).

	LIVER		PANCREAS		MUSCLE	
	Tgl173 *vs.* Con	Tgl178 *vs.* Con	Tgl173 *vs.* Con	Tgl178 *vs.* Con	Tgl173 *vs.* Con	Tgl178 *vs.* Con
Pearson Corr.[1]		0.116		0.309		0.513
Pearson Corr.[2]				0.196 Liver		0.139 Pancreas
						0.334 Liver
Regression		0.656		0.226		0.192
(*F* significance)				0.264 Liver		0.606 Pancreas
						0.205 Liver

Pearson Corr.[1] denotes gene expression correlation within tissue amongst the two Tg lines, and Pearson Corr.[2] across tissues (n=4 per mouse genotype).
Regression analysis (*F* significance) refers to genes studied in either Tg lines or tissues.
